# The SLC34A2-ROS-HIF-1-induced up-regulation of EZH2 expression promotes proliferation and chemo-resistance to apoptosis in colorectal cancer

**DOI:** 10.1042/BSR20180268

**Published:** 2019-05-21

**Authors:** Xu Li, Junjie Xing, Hantao Wang, Enda Yu

**Affiliations:** Department of Colorectal Surgery, Changhai Hospital, Second Military Medical University, Shanghai 200433, People’s Republic of China

**Keywords:** colorectal cancer, chemo-resistance, EZH2, HIF-1α, ROS, SLC34A2

## Abstract

Growing evidence has uncovered that *SLC34A2* plays an evident role in the progression in several types of tumors. However, the biological function and underlying molecular mechanisms of *SLC34A2* remain largely unknown. Here, we indicated that SLC34A2 expression was markedly increased in SW480 and HT29 cell line cells compared with that in normal colorectal epithelial cell line cells. Array analysis displayed that the expression of enhancer of zeste 2 (EZH2) decreased considerably when *SLC34A2* was knocked down. We demonstrated that SLC34A2 induced EZH2 expression and activated its promoter activity. Serial 5′ deletion and site-directed mutagenesis revealed that the induction of EZH2 expression by SLC34A2 was dependent upon the hypoxia-inducible factor 1 (HIF-1)-2 binding site directly within EZH2 promoter. Moreover, HIF-1 activation was proved essential for SLC34A2-induced EZH2 expression. Reactive oxygen species (ROS) generation contributed to the stabilization of HIF-1α by leading to the binding of HIF-1α to the EZH2 promoter, which resulted in increased EZH2 expression. Additionally, we showed that the inhibition of both HIF-1α expression and ROS generation by YC-1 or BHA, respectively, decreased SLC34A2-induced EZH2 overexpression. Significantly, SLC34A2-induced EZH2 overexpression promoted the proliferation and chemo-resistance to apoptosis in colorectal cancer (CRC) cells *in vitro* and *in vivo*. Altogether, we conclude that the SLC34A2-ROS-HIF-1-induced overexpression of EZH2 promotes CRC cells proliferation and chemo-resistance to apoptosis. SLC34A2-ROS-HIF-1-EZH2 signaling pathway might serve as a novel therapeutic target against CRC.

## Introduction

Colorectal cancer (CRC) is one of the most common and fatal neoplastic diseases worldwide [1]. Although improved treatment strategies have increased the overall survival rates in the early stages, 5-year survival rates have not raised substantially in the past decades [2]. Most CRC patients with distant metastasis are not suitable candidates for conventional intervention. Meanwhile, the current chemotherapeutic drugs of non-surgical treatment, including cisplatin, 5-fluorouracil, capecitabine, and doxorubicin, which are used against advanced CRC are largely unsuccessful [3,4]. Thus, from a therapeutic perspective, defining the molecular mechanisms underlying the progression and chemo-resistance of CRC may contribute to decreasing morbidity and mortality.

*SLC34A2*, belonging to the solute carrier gene family, encodes the type II Na/Pi co-transporter (NaPi2b) [5]. NaPi2b is a multitransmembrane sodium-dependent phosphate transporter responsible for transcellular inorganic phosphate absorption [6]. An increased expression of NaPi2b has been reported in ovarian cancer [7], breast cancer [8,9], lung cancer [10], and gastric cancer [11]. It was found that SLC34A2 contributed to the maintaining of stem cell-like phenotypes in breast cancer and non-small-cell lung cancer [10]. Furthermore, Ge et al. [8] revealed that SLC34A2 induced chemo-resistance in CD44^+^ CD24^−^ breast cancer stem cell-like cells via Bmi1-ABCC5 signaling, which implies that SLC34A2 may be included in chemo-resistance in breast cancer. Nevertheless, the expression level of SLC34A2 (NaPi2b) in CRC is poorly understood. Additionally, the biological function of SLC34A2 and its underlying mechanisms in CRC remain unclear.

Recently, some studies identified a chromosomal SLC34A2-ROS1 rearrangement in gastric cancer and lung adenocarcinoma [12,13]. It was found that patients who had SLC34A2-ROS1 transcripts had poorly differentiated histology with recurrence and death within 2 years of curative surgery [12]. Reactive oxygen species (ROS) are largely produced by the mitochondria, and they have been shown to mediate the activation of signaling cascades that are needed to regulate growth, differentiation, proliferation, and apoptosis [14,15]. Several recent studies revealed that a burst of mitochondrial ROS led to hypoxia-inducible factor 1 (HIF-1) α (HIF-1α) stabilization under normoxia [16,17]. The transcription factor HIF-1 is a global regulator of O_2_ homeostasis and the adaptation to O_2_ deprivation [18]. HIF-1 is composed of α and β subunits [18]. Hypoxia-inducible factor 1α (HIF-1α), conferring protection against cell death, is induced by hypoxia and plays an adaptive role [19]. Elevated HIF-1α expression was reported linking with tumor metastasis, resistance to therapy, and poor survival [20]. Although current research supports a role for HIF-1 as a prominent regulator of the genetic response to hypoxia, it has recently been shown that some other factors, like certain pro-inflammatory cytokines, are able to stabilize HIF-1α and activate HIF-1 [14,15]. However, the relationship between SLC34A2 and HIF-1 in CRC is unclear.

Here, we aim to investigate the biological function and underlying molecular mechanisms of SLC34A2. Our data indicated that SLC34A2 expression was markedly increased in SW480 and HT29 cell line cells compared with that in normal colorectal epithelial cell line (CCD 841C_O_N) cells. Array analysis displayed that the expression of enhancer of zeste 2 (EZH2) decreased considerably when SLC34A2 was knocked down. We demonstrated that SLC34A2 induced EZH2 expression and activated its promoter activity. Serial 5′ deletion and site-directed mutagenesis revealed that the induction of EZH2 expression by SLC34A2 was dependent upon the HIF1-2 binding site directly within EZH2 promoter. Moreover, HIF-1 activation was proved essential for SLC34A2-induced EZH2 expression. ROS generation contributed to the stabilization of HIF-1α by leading to the binding of HIF-1α to the EZH2 promoter, resulting in increased EZH21 expression. Additionally, we showed that the inhibition of both HIF-1α expression and ROS generation by YC-1 or BHA, respectively, decreased SLC34A2-induced EZH2 overexpression. Significantly, SLC34A2-induced EZH2 overexpression promoted the proliferation and chemo-resistance to apoptosis in CRC cells *in vitro* and *in vivo*.

## Materials and methods

### Animal and cell culture

Female athymic BALB/c nu/nu mice, 3–4 weeks old, obtained from HFK Bioscience (China), were maintained at the Animal Core Facility at Changhai Hospital, Second Military Medical University, under specific pathogen-free (SPF) condition. All studies on mice were conducted in accordance with the National Institutes of Health ‘Guide for the Care and Use of Laboratory Animals’ and were approved by the ethical committee of Changhai Hospital, Second Military Medical University.

Human colorectal cell lines SW480 and HT29 were purchased from CLS Cell Lines Service GmbH (Eppelheim, Germany). The cell line was incubated in Dulbecco’s modified Eagle’s medium supplemented with d-glucose 4500 mg/l, 4 mM glutamine, and 110 mg/l sodium pyruvate, 10% FBS, 1× penicillin–streptomycin and non-essential amino acids (all purchased from Gibco-Invitrogen, Waltham, MA, U.S.A.).

### Quantitative real-time RT-PCR analysis

Total RNA was extracted using TRIzol reagent (Invitrogen, U.S.A.) according to the manufacturer’s protocol in the condition of low temperature. Synthesis of cDNA with reverse transcriptase was performed by PrimeScript RT reagent Kit Perfect Real Time (TaKaRa, China). For gene(s) expression analysis, quantitative real-time PCR analysis was done using the iCycler iQ5 real-time PCR Detection system (Bio-Rad, U.S.A.) with SYBR Green Reagents (Bio-Rad, U.S.A.). β-actin was amplified as an internal control. Comparative gene expression analysis was performed using the 2^−ΔΔ*C*^_t_ method with normalization to the level of internal control β-actin.

### Luciferase reporter assay

The luciferase activity was detected with the Dual Luciferase Assay (Promega, U.S.A.) according to the manufacturer’s instructions. The transfected cells were lysed in the culture dishes with a lysis buffer, and the lysates were centrifuged at maximum speed for 1 min in an Eppendorf microcentrifuge. The relative luciferase activity was determined by a Modulus TM TD20/20 Luminometer (Turner Biosystems, U.S.A.), and the transfection efficiency was normalized by *Renilla* activity.

### Lentiviral transduction

SW480 and HT29 cells dissociated were spin-infected with 1 ml of p-*SLC34A2* (Shanghai GenePharma Co. Ltd, China) or *SLC34A2*-si-RNA lentiviral knockdowns (Shanghai GenePharma Co. Ltd, China) packbag (the sequence for si-SLC34A2: 5′-CTCCCTGGATATTCTTAGTTT-3′). For both systems, cells were infected with lentiviral media at a multiplicity of infection (MOI) of 40, in the presence of 8 mg/ml polybrene (Sigma–Aldrich, U.S.A.), overnight in a 37°C incubator. The transduction efficiency of both p-*SLC34A2* and *SLC34A2*-si-RNA evaluated by GFP expression and quantitative real-time RT-PCR analysis (qPCR). Stable clones of *SLC34A2*-si-RNA (vector: pHHsi-hU6-GFP-Puro) were selected using puromycin. After detecting the efficiency of si-*SLC34A2* stable cell construction (methods as qPCR part), we used p-*SLC34A2* to infect si-*SLC34A2* stable cells repeatedly, while stable clones of p-*SLC34A2* (vector: pcDNA3.1) were selected using G418.

### Western blot

The different treated cells were washed with ice-cold PBS and then lysed by protein lysate with RIPA buffer (Pierce, U.S.A.). After centrifugation at 12900 ***g*** for 10 min at 4°C, the protein concentration was measured by BCA protein assay kit (Pierce, U.S.A.). Then, all proteins were resolved on a 10% SDS denatured polyacrylamide gel and were then transferred on to a PVDF membrane (Millipore, U.S.A.). Membranes were incubated with blocking buffer for 60 min at room temperature and were then incubated with a specific primary antibody with Blotto overnight at 4°C. The membranes were washed and incubated with a horseradish peroxidase (HRP)–conjugated secondary antibody. Blots were then exposed to secondary antibodies and visualized by an ECL blotting analysis system (GE Healthcare Life Sciences, China).

### MTT assay

Cells were seeded in 96-well microtiter plates. At 48 h, the supernatants were aspirated, and MTT was added to each well. After a 3-h incubation at 37°C, DMSO was added to dissolve the formazan crystals, and the absorbance was measured at 490 nm using a Microplate Reader Model 680.

### Measurement of intracellular ROS

The intracellular ROS were measured using Cellular Reactive Oxygen Species Detection Assay Kit (Abcam, U.S.A.). The cells adhering to the coverslip were incubated with 20 µM ROS Red Stock Solution at 37°C in the dark for 30 min. Hoechst 33342 was added at a final concentration of 1 µM to the ROS Red Stock Solution staining solution during the last 5 min of the incubation. The cells were then washed in PBS and analyzed by confocal microscope.

### HIF-1 binding and ChIP assay

Cells that had been transfected with relative plasmids were cross-linked using 1% formaldehyde at 37°C for 10 min. After washing with PBS, the cells were resuspended in 300 µl of lysis buffer (50 mM Tris, pH 8.1), 10 mM EDTA, 1% SDS, and 1 mM PMSF). DNA was sheared to small fragments by sonication. The supernatants were precleared using a herring sperm DNA/protein G-Sepharose slurry (Sigma–Aldrich, U.S.A.). For the binding and competition assay, nuclear extracts from cells were incubated with the wild-type (WT) or mutated HIF1-2 oligonucleotides. The immunoprecipitated DNA was retrieved from the beads with 1% SDS and a 1.1 M NaHCO_3_ solution at 65°C for 6 h. DNA was then purified using a PCR purification kit (Qiagen, U.S.A.). The primers 5′-GGAGCAGAGGAGCCTGAG-3′ and 5′-GCGAGCCGAGGGAGAGTT-3′ were used to amplify the HIF1-2 binding site. The primers 5′-CGGAATGCCGAGACAAGG-3′ and 5′-TGGCACCG- GAGCTTTCAGTT-3′ were used to examine the HIF1-2 binding site. The primers 5′-CTGGAGTGCAGTGGTGTGAT-3′ and 5′-GCGAAACCG-CTTCTCTACTA-3′, which did not contain HIF-1 binding sites, were used as a negative control (NC). As for the ChIP assay, the recovered supernatants were incubated with a specific antibody or an isotype control (IgG) for 2 h in the presence of herring sperm DNA and protein G-Sepharose beads. The immunoprecipitated DNA was retrieved from the beads with 1% SDS and a 1.1 M NaHCO3 solution at 65°C for 6 h. DNA was then purified using a PCR purification kit (Qiagen, U.S.A.). The primers were detailed as above.

### TUNEL assay

TUNEL staining was performed by the DeadEnd™ Fluorometric TUNEL system according to the manufacturer’s instructions (Promega, U.S.A.) in xenograph tumor tissues derived from si-*NC*, si-*SLC34A2* and si-*SLC34A2* + p*-EZH2* cells. Xenograph tumor cells were then observed under a fluorescence microscope (Olympus Optical Co., Germany), and a nucleus with bright green fluorescence staining was recorded as a TUNEL-positive event.

### Annexin V staining and FACS analysis

Apoptosis was measured using the Annexin V-FITC apoptosis detection kit (Bender Medsystems, Austria). Cells were collected and resuspended in binding buffer for analysis. Cells were also stained with propidium iodide to detect dead cells. Apoptotic cells were measured by LSR II FACS, and data analysis were performed with the standard Cell Quest software.

### Statistical analysis

All the assay results represent the arithmetic mean ± S.D. standard error of triplicate determinations of at least three independent experiments done under the same conditions. The significant difference of the experimental results was calculated using one-way ANOVA and an unpaired Student’s *t* test with SPSS13.0 software. Significances were ***, *P*<0.001; **, *P*<0.01; *, *P*<0.05.

## Results

### SLC34A2 induces EZH2 expression and activates EZH2 promoter activity in CRC cells

**Figure 1:**
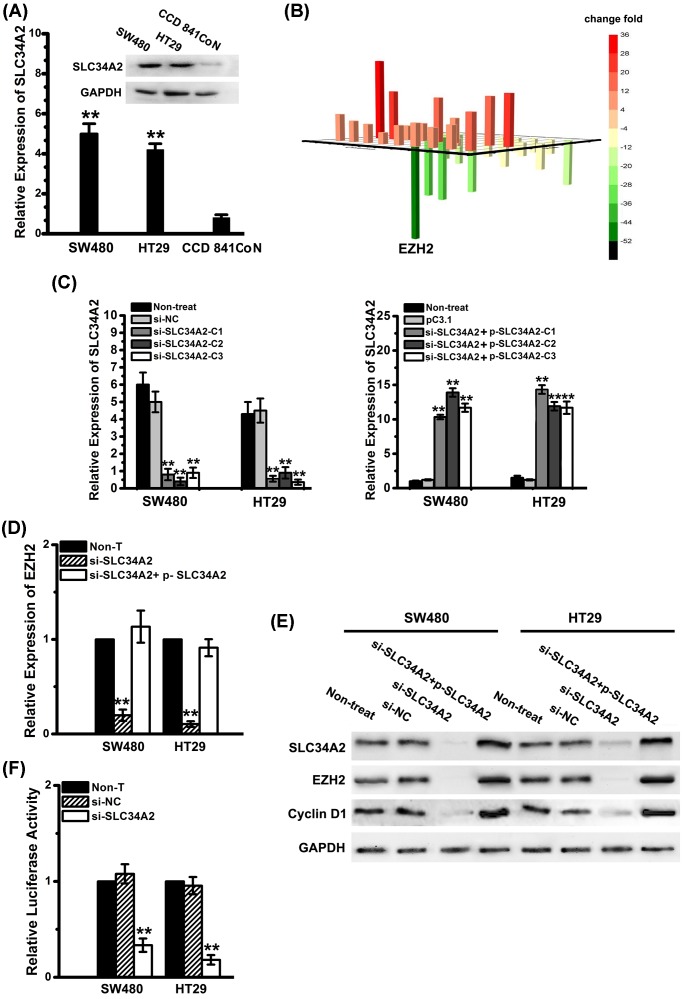
SLC34A2 induces EZH2 expression and activates EZH2 promoter activity (**A**) Relative expression of SLC34A2 (band size: 76 kDa) in different cell line was examined by qPCR and Western blot. Columns, mean of three individual experiments; S.D., **, *P*<0.01. All data are representative of at least three independent experiments. (**B**) The proliferation relative gene expression levels are represented in the 3D bar chart. For each gene there are four replicates in this assay. All data are representative of at least three independent experiments. (**C**) Relative expression of SLC34A2 in constructed stable sublines of SW480 and HT29 by infection with lenti-si-SLC34A2 or lenti-p-SLC34A2 was examined by qPCR. For each gene, there are six replicated wells in qPCR. Columns, mean of three individual experiments; S.D., **, *P*<0.01. (**D**) Relative expression of EZH2 was examined by qPCR. For each gene there are six replicated wells in qPCR. Columns, mean of three individual experiments; S.D., **, *P*<0.01. (**E**) Western blot was used to detect the protein levels of SLC34A2, EZH2 (band size: 85 kDa) and cyclin D1 (band size: 34 kDa) expression in lenti-si-SLC34A2, lenti-si-SLC34A2+ p-SLC34A2 or non-T group. GAPDH was used as a loading control. All data are representative of at least three independent experiments. (**F**) The luciferase activity was determined in lenti-si-SLC34A2, lenti-si-NC, and non-T group. There are six replicated wells for each group in this assay. Columns, mean of three individual experiments; S.D., **, *P*<0.01. Abbreviations: lenti, lentivirus mediated infection; non-T, non-treated; p, pcDNA3.1.

By qPCR and Western blot assays, we uncovered that the expression of SLC34A2 in SW480 and HT29 cell line cells was markedly increased compared with that in CCD 841C_O_N cell line cells (the human normal colorectal epithelial cell) (Figure 1A).

To explore the underlying mechanisms of SLC34A2 in CRC cells, we employed array analysis of human tumor proliferation genes after knockdown *SLC34A2* CRC cells. The assay result indicated that the expression of EZH2 decreased considerably when *SLC34A2* was knocked down (Figure 1B). To testify the result above and further detect the connection between SLC34A2 and EZH2, we constructed stable sublines of SW480 and HT29 by infection with lenti-si-*SLC34A2* or lenti-p-*SLC34A2* (Figure 1C). SW480 and HT29 infected with lenti-si-*SLC34A2* showed markedly decreased level of EZH2, while si-*SLC34A2* cells repeatedly infected with lenti-p-*SLC34A2* displayed restored level of EZH2 (Figure 1D,E). Also, the infection with lenti-si-*SLC34A2* decreased the expression of *cyclin D1*, which is the target gene of EZH2 (Figure 1E).

To further determine whether SLC34A2 induced EZH2 expression is regulated by the transactivation of its promoter, SW480 and HT29 cells were transfected with a reporter plasmid containing the promoter of *EZH2* gene. The *EZH2* promoter activity was markedly decreased after the infection with si-*SLC34A2* (Figure 1F), suggesting that the activation of the *EZH2* promoter is dependent on *SLC34A2*.

### HIF-1 binding site is indispensable for SLC34A2-induced EZH2 activation

**Figure 2:**
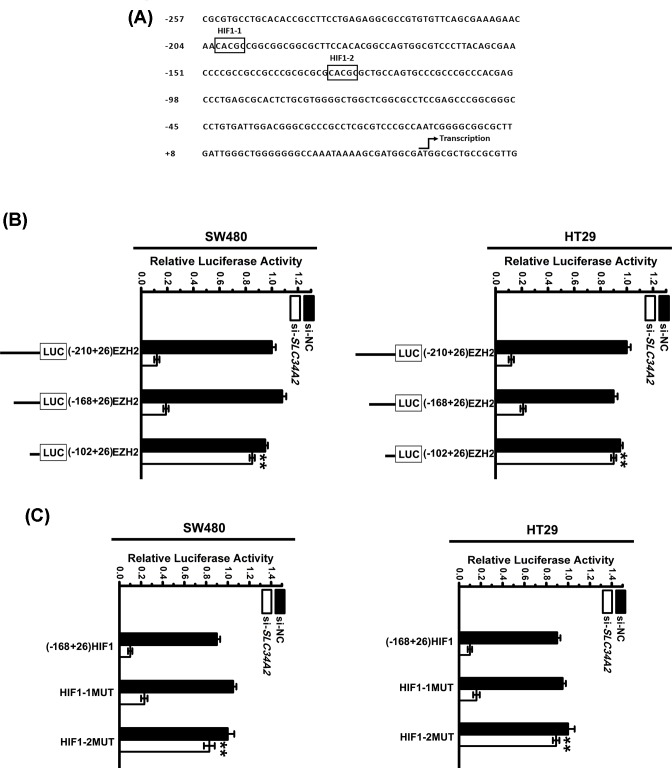
HIF-1 binding site is indispensable for SLC34A2-induced EZH2 activation (**A**) A sequence analysis of the 5′-flanking region of the human EZH2 promoter revealed two putative HIF-1 binding sites. The transcription start site is also indicated in the figure. (**B**) On the left, a schematic representation of the reporter gene constructs is shown. On the right, the bar graphs represent the relative levels of luciferase activity in si-NC and si-SLC34A2 infected samples. Columns, mean of three individual experiments; S.D., **, *P*<0.01. There are six replicated wells for each group in this assay. (**C**) Cells were transfected with one of the different mutated EZH2 promoter constructs or the WT-EZH2 promoter, and the cells were then infected with or without si-SLC34A2. The luciferase activity was measured. Columns, mean of three individual experiments; S.D., **, *P*<0.01. There are six replicated wells for each group in this assay. All data are representative of at least three independent experiments.

To investigate the roles of the *cis*-elements of EZH2 promoter in SLC34A2-induced *EZH2* gene transcription, we constructed reporter containing serial 5′ deletions of the EZH2 promoter and transfected them into SW480 and HT29 cells. Analysis of the *cis*-regulatory elements in the EZH2 promoter between nt-210 and nt-102 revealed two potential HIF-1 binding sites: HIF1-1 and HIF1-2 (Figure 2A). It was found that the deletion between nt-168 and nt-102 could not markedly reduce the EZH2 expression when the *SLC34A2* was knocked down, whereas in the same condition deletions between nt-210 and nt-168 could (Figure 2B). At the meantime, both potential HIF1 binding sites on EZH2 promoter were mutated to determine the biological function of HIF1 binding site in SLC34A2-induced EZH2 activation. Transfected the mutated constructs into SW480 and HT29 cells for 24 h before lenti-si-*SLC34A2* infected these cells. The mutation of HIF1-2 significantly increased the activation of EZH2 promoter by si-*SLC34A2* infection compared with that without any mutation (Figure 2C), indicating that HIF1-2 binding site might be involved for SLC34A2-induced EZH2 activation.

### HIF-1 directly binds to the EZH2 promoter

**Figure 3:**
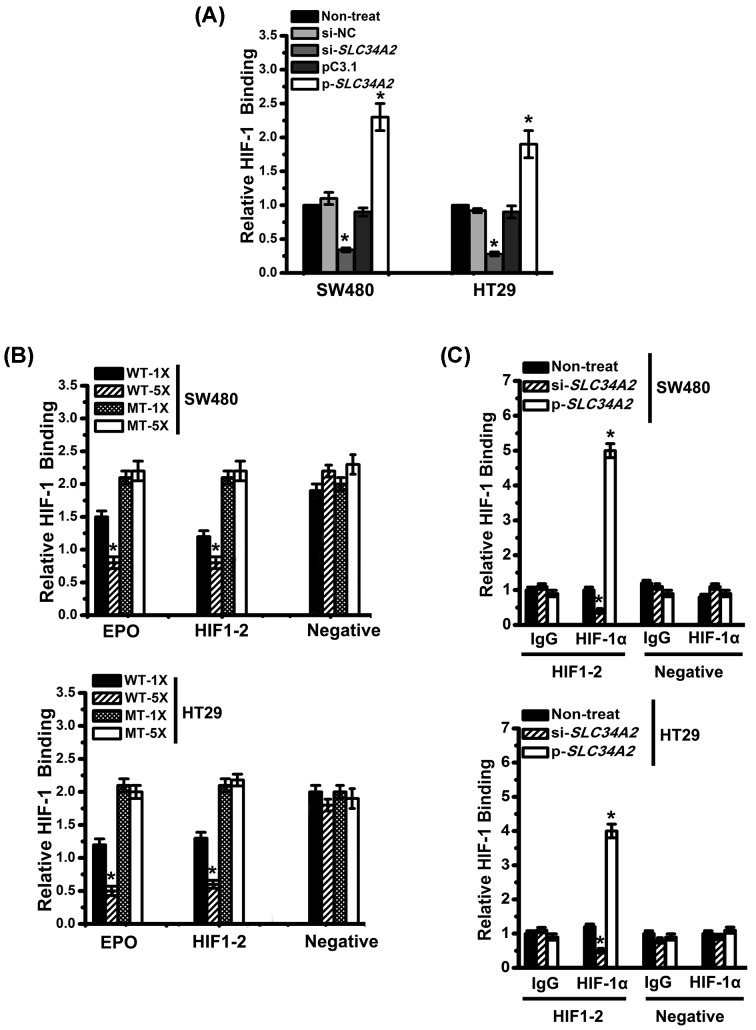
HIF-1 directly binds to the EZH2 promoter (**A**) The HIF-1α DNA binding activity was measured in different groups. Columns, mean of three individual experiments; S.D., *, *P*<0.05. All data are representative of at least three independent experiments. (**B**) The 20 pmol (1×) or 100 pmol (5×) of WT or the MT-EPO promoter hypoxia-responsive element oligonucleotides or the EZH2 promoter hypoxia-responsive element oligonucleotides were used to determine the HIF-1α DNA binding activity. Columns, mean of three individual experiments; S.D., *, *P*<0.05. (**C**) ChIP assays were performed using an antibody to HIF-1α or a control antibody to pull down the DNA fragment containing the HIF1-2 binding site on the EZH2 promoter in different infection group. A distant region of EZH2 promoter that do not contain putative HIF-1 binding site served as a NC. Columns, mean of three individual experiments; S.D., *, *P*<0.05. All data are representative of at least three independent experiments.

We used HIF1-2 binding and competition ELISAs to further determine whether HIF1-2 binds to the EZH2 promoter. The HIF1-2 DNA binding activity was measured after infection with si-*SLC34A2* or p-*SLC34A2* for 48 h. The HIF-1 DNA binding activity was significantly decreased in cells infected with si-*SLC34A2* compared with that in untreated cells or cells infected with si-NC (Figure 3A). However, the HIF1-2 DNA binding activity increased in p-*SLC34A2* group (Figure 3A). These data suggested that SLC34A2 positively regulated the HIF-1 DNA binding activity in CRC cells. Next, the pretreatment of nuclear extracts with WT oligonucleotides of HIF1-2 significantly abolished HIF-1 binding to the HIF-1-binding oligonucleotides of EPO, on the other hand, the mutant (MT) HIF1-2 oligonucleotides did not decrease the HIF-1 binding activity (Figure 3B). In addition, increased binding activity was observed with the addition of increasing amounts of nuclear extracts. ChIP assays were also used to confirm the binding of HIF-1 to the EZH2 promoter. Control reactions (IgG isotype) did not exhibit specific immunoprecipitation. However, reactions containing the HIF-1α antibody displayed specific binding to HIF1-2 binding site on the EZH2 promoter (Figure 3C). Taken together, these results suggest that HIF-1 directly binds to the EZH2 promoter.

### HIF-1 activation is essential for SLC34A2-induced EZH2 expression

**Figure 4:**
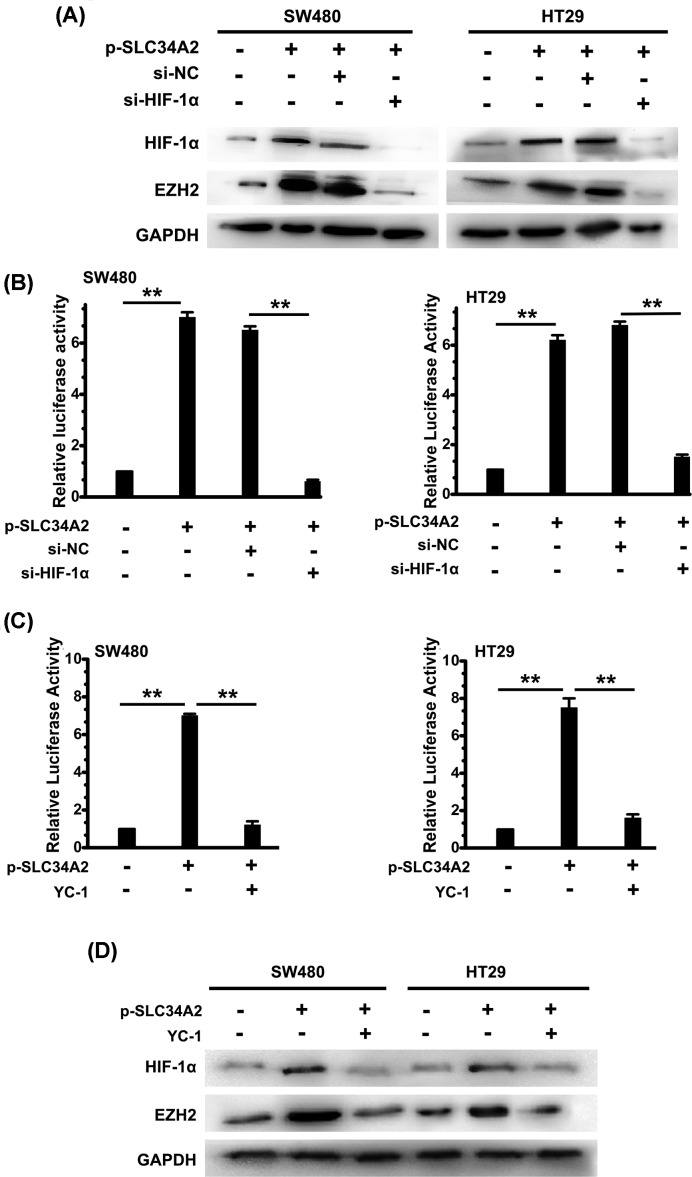
HIF-1 activation is essential for SLC34A2-induced EZH2 expression (**A**) Cells were infected with HIF-1α siRNA or control siRNA and then infected with or without p-SLC34A2. The protein levels of EZH2 and HIF-1α (band size: 110 kDa) were measured by Western blot. GAPDH was used as a loading control. All data are representative of at least three independent experiments. (**B**) Luciferase activity was assayed in cells infected with p-SLC34A2, si-HIF-1α, or si-NC. Columns, mean of three individual experiments; S.D., **, *P*<0.01. There are six replicated wells for each group in this assay. (**C**) Luciferase activity was assayed in cells infected with p-SLC34A2 or treated with YC-1. Columns, mean of three individual experiments; S.D., **, *P*<0.01. There are six replicated wells for each group in this assay. (**D**) Cells were infected with p-SLC34A2 or treated with YC-1. The protein levels of EZH2 and HIF-1α were measured by Western blot. GAPDH was used as a loading control. All data are representative of at least six independent experiments. Abbreviation: p, pcDNA3.1.

First, we investigated the effect of SLC34A2 on HIF-1α expression. The lentivirus method was used to knockdown HIF-1α expression in SW480 and HT29 cells. The specificity of the lenti-si-*HIF-1α* was also confirmed (Figure 4A). p-*SLC34A2* infection increased the expression of HIF-1α and EZH2, indicating that SLC34A2 lies on the upstream of HIF-1α and EZH2. The protein level of EZH2 was enhanced in p-*SLC34A2* group, while si-*HIF-1α* infection resulted in EZH2 expression significantly declined in p-*SLC34A2* group (Figure 4A). These data imply that SLC34A2 stabilizes the protein level of HIF-1α that is required for SLC34A2-induced EZH2 expression. Second, we investigated the functional role of HIF-1α on SLC34A2-induced EZH2 expression. The luciferase reporter assay revealed that si-HIF-1α significantly inhibited the SLC34A2-mediated transactivation of the EZH2 promoter (Figure 4B), which was further in-line with the result of Western blot assay (Figure 4A). At the meantime, YC-1, a HIF-1α inhibitor, was used to reduce the expression of HIF-1α induced by SLC34A2. We showed that YC-1 abolished the SLC34A2-mediated activation of the EZH2 promoter and blocked SLC34A2-mediated EZH2 overexpression (Figure 4C,D).

### SLC34A2-induced HIF-1 activation and EZH2 expression are ROS dependent

**Figure 5:**
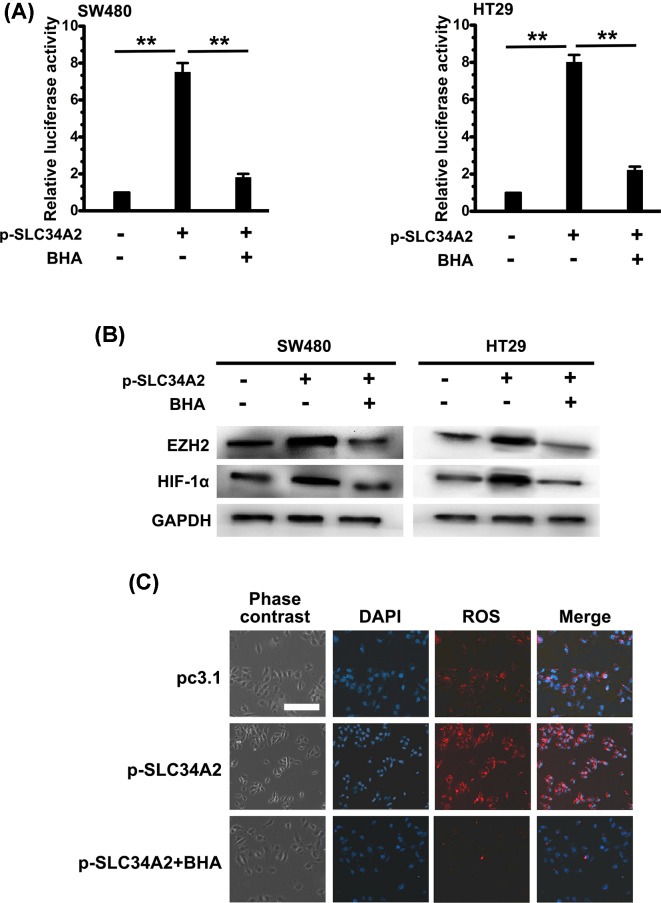
SLC34A2-induced HIF-1 activation and EZH2 expression are ROS dependent (**A**) Luciferase activity was assayed in cells infected with p-SLC34A2 or treated with BHA. Columns, mean of three individual experiments; S.D., **, *P*<0.01. (**B**) Cells were infected with p-SLC34A2 or treated with BHA. The protein levels of EZH2 and HIF-1α were measured by Western blot. GAPDH was used as a loading control. All data are representative of at least three independent experiments. (**C**) Cells were infected with or without p-SLC34A2 in the presence of BHA for 24 h. The cells were then staining with Cellular Reactive Oxygen Species Detection Assay Kit. Scale bar, 100 µm. All data are representative of at least three independent experiments. Abbreviation: p, pcDNA3.1.

Several studies have demonstrated that HIF-1α stabilization is regulated by ROS [16]. So we examine the inhibitory effect of the antioxidant BHA on EZH2 expression in SW480 and HT29 cells to investigate if ROS is involved in EZH2 activated by SLC34A2. SW480 and HT29 cells which were transfected with EZH2 reporter plasmids were treated with BHA. As it was displayed that the inhibition of ROS production by BHA reduced the EZH2 promoter activity in the luciferase reporter assay (Figure 5A). Additionally, to confirm the role of ROS in EZH2 expression, lenti-p-*SLC34A2* cells were treated with BHA. The BHA treatment significantly decreased HIF-1 α and EZH2 expression induced by SLC34A2 (Figure 5B). At the meanwhile, the induction of intracellular ROS by SLC34A2 was confirmed by confocal microscope (Figure 5C). These data indicate that SLC34A2-induced EZH2 expression by HIF-1 activation are dependent on ROS.

### SLC34A2 promotes the proliferation of CRC cells and their cisplatin resistance to apoptosis

**Figure 6:**
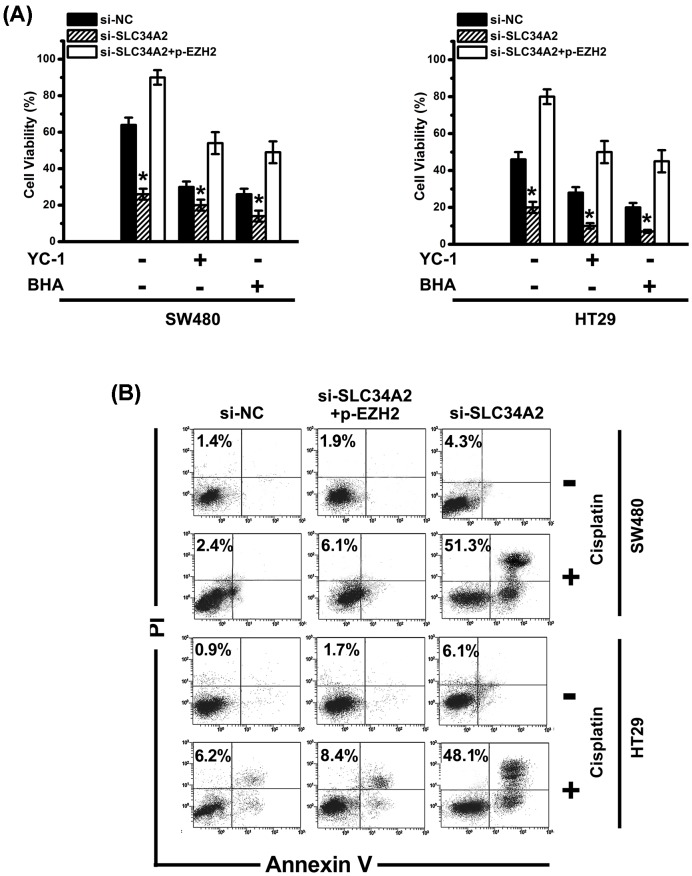
SLC34A2 promotes the proliferation of CRC cells and their cisplatin resistance to apoptosis *in vitro* (**A**) Cell viability of si-NC, si-*SLC34A2*, si-*SLC34A2* + p-*EZH2* cells with or without YC-1 or BHA were determined by MTT. Columns, mean of three individual experiments; S.D., *, *P*<0.05. (**B**) With or without cisplatin, the percentage of Annexin V^+^PI^+^/Annexin V^+^PI^−^cells was measured by flow cytometry. All data are representative of at least three independent experiments. Abbreviation: p, pcDNA3.1.

We demonstrated that EZH2 expression was induced by SLC34A2-ROS-HIF-1 signaling pathway as above. To detect the biological function on proliferation and chemo-resistance of up-regulated EZH2 induced by SLC34A2, we performed following assays. First, cell viability was tested. Cell viability of si-*SLC34A2* cells was significantly reduced, while the co-infection with p-*EZH2* and si-*SLC34A2* reversed the cell viability (Figure 6A). YC-1 or BHA treatment further declined the growth of SW480/HT29 cells (Figure 6A). On the other hand, the inhibition of cell growth YC-1/BHA could be reversed by p-*EZH2* (Figure 6A). Additionally, in the presence of cisplatin, the Annexin-V/PI staining assay displayed that the inhibition of EZH2 expression by si-*SLC34A2* increased the apoptosis (Figure 6B). Nevertheless, cells infected with p-*EZH2* were resistant to apoptosis (Figure 6B).

**Figure 7:**
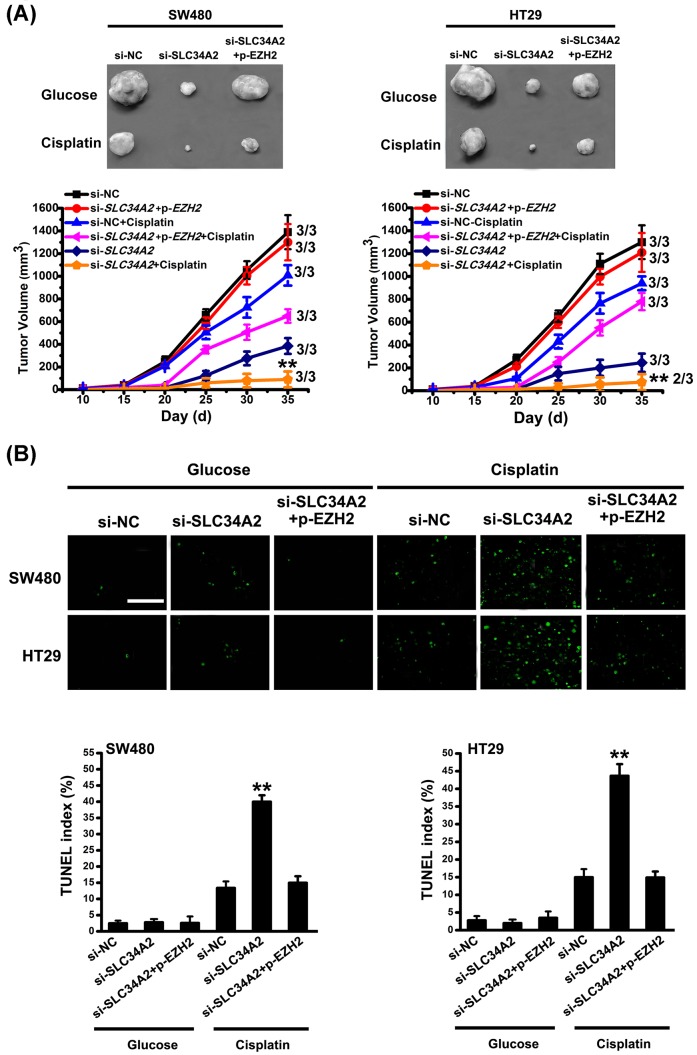
SLC34A2 promotes the proliferation of CRC cells and their cisplatin resistance to apoptosis *in vivo* (**A**) The potential of tumor initiation of fractions, si-NC, si-*SLC34A2*, si-*SLC34A2* + p-*EZH2* cells were tested by subcutaneous injection, and representative tumor volumes were measured following treatment with or without three cycles of cisplatin. Columns, mean of three individual experiments; S.D., **, *P*<0.01. The number of mice for each cohort used is three. (**B**) Apoptosis in xenograft tumors derived from si-NC, si-*SLC34A2*, si-*SLC34A2* + p-*EZH2* cells following treatment with or without three cycles of cisplatin were detected by TUNEL assay. There are three replicated slices for each tissue group in this assay. Scale bar, 100 µm. Columns, mean of three individual experiments; S.D., **, *P*<0.01. Abbreviation: p, pcDNA3.1.

*In vivo*, we found that, in the glucose group, xenograft tumors derived from si-*SLC34A2* cells were markedly smaller than those from si-NC cells, whereas co-infected with p-*EZH2* and si-*SLC34A2* resulted in restored tumor volume (Figure 7A). Upon intratumor injection with cisplatin, the size of xenograft tumors in co-infection group with p-*EZH2* and si-*SLC34A2* abolished the reduction in xenograft tumors resulting from si-*SLC34A2* infection alone (Figure 7A). Similarly, co-infection group with p-*EZH2* and si-*SLC34A2* relieved the higher TUNEL index due to si-*SLC34A2* infection alone (Figure 7B). Taken together, these results suggest that the SLC34A2-induced up-regulation of EZH2 expression promotes the proliferation of CRC cells and their cisplatin resistance to apoptosis.

## Discussion

Proliferation and chemo-resistance, one of the crucial hallmarks of cancer, is the major cause of death in patients with cancers, especially in CRC. Thus, it is urgent to identify the factors involved in proliferation and chemo-resistance and understand the underlying molecular mechanisms.

In the present study, we acquired both *in vitro* and *in vivo* evidence supporting the critical role of SLC34A2 in the induction of ROS-HIF-1-EZH2 signaling pathway in the proliferation and chemo-resistance of CRC, suggesting that SLC34A2 inhibition supplies a potential therapeutics in CRC. This is the first investigation to directly analyze the biological function of SLC34A2 and its underlying mechanisms in CRC. We demonstrated that SLC34A2 induced EZH2 expression and activated EZH2 promoter activity in CRC cells. EZH2 facilitates repression of its target genes via trimethylation of lysine residue 27 on histone 3 (H3K27me3) [21]. The oncogenic function of *Ezh2* has been linked to both PRC2-dependent and PRC2-independent activities [22,23]. A high EZH2 expression has been reported being associated with poor disease outcome in different types of tumors, like breast, prostate, and pancreatic cancers [23–25]. In CRC, EZH2 demonstrated up-regulation and linking with proliferation and metastasis [26]. However, the upstream mechanisms are blurred. Here, our data strongly suggest that the activation of the EZH2 promoter is dependent on SLC34A2. Identifying the regulatory mechanism that underlies EZH2 overexpression in CRC is crucial for further understanding the tumorigenic process and the development of novel approaches for cancer therapy.

HIF-1, a transcription factor, is activated during hypoxia and is involved in cell proliferation and apoptosis [27]. Several studies have displayed that HIF-1 binding to the promoter of target genes to regulate target genes’ transcription is crucial to tumorigenesis and progression [18,20]. HIF-1α is induced by hypoxia conferring protection against cell death an adaptive role [19]. HIF-1α regulates the transcription of multiple genes in tumor cells after binding to its cognate enhancer sequence in the promoters of its target genes [20]. Our results revealed a direct relationship between the level of HIF-1α and EZH2 by the luciferase reporter assay. The mutation of HIF1-2 decreased luciferase activity, suggesting that HIF-1 binding site is involved in SLC34A2-induced EZH2 activation. Mahara et al. [28] found that HIF-1-α was a crucial modulator of PRC2 and EZH2 function in breast cancer. However, how HIF-1-α affects EZH2 expression, directly or indirectly, is unrevealed. In our study, HIF-1 binding and competition assays further determined that HIF-1 was a critical mediator of SLC34A2-induced EZH2 expression. Additionally, ChIP assays were used to confirm that HIF-1 directly bond to the EZH2 promoter.

Recently, several studies uncovered that a burst of mitochondrial ROS stabilized HIF-1α under normoxia [17]. Here, the antioxidant BHA was used to block the generation of ROS to investigate the connection between SLC34A2, ROS generation and HIF-1α stabilization in CRC cells. Western blot and luciferase assays showed that inhibiting ROS generation significantly reduced the expression of HIF-1α and EZH2, suggesting that SLC34A2-induced EZH2 expression by HIF-1 activation are dependent on ROS in CRC cells.

Considering that the biological functions and underlying mechanisms of SLC34A2 in CRC is poorly understood, we, here, demonstrated that SLC34A2-induced up-regulation of EZH2 expression promoted the proliferation of CRC cells and their cisplatin resistance to apoptosis *in vivo* and *in vitro*, which implied that EZH2 also could serve as the target of potential therapeutics. In the meantime, YC-1 or BHA treatment further declined the growth of SW480/HT29 cells. On the other hand, the inhibition of cell growth YC-1/BHA could be reversed by p-*EZH2.* These data imply that each element in SLC34A2-ROS-HIF-1-EZH2 signaling pathway contributes to the proliferation of cisplatin resistance to apoptosis in CRC.

In conclusion, we demonstrated that the SLC34A2-ROS-HIF-1-induced overexpression of EZH2 promoted CRC cells proliferation and chemo-resistance to apoptosis. Therefore, this investigation of the mechanism underlying SLC34A2 in CRC sheds new light on development of potential therapeutics against CRC via targetting SLC34A2-ROS-HIF-1- EZH2 signaling pathway.

## Supporting information

**Supplementary Figure S1:** The expression of EZH2 and HIF-1α were detected by western blot in SW480 and HT29 cells infected with si-*HIF*-1α or si-NC. GAPDH was used as a loading control.

**Supplementary Figure S2:** The effect of YC-1 and BHA were tested on SW480 and HT29 cells. (A) Luciferase activity was assayed in cells treated with or without YC-1 or BHA. Note: Columns, mean of three individual experiments; SD, **, P < 0.01. (B) SW480 cells were treated with or without YC-1 or BHA. The protein levels of EZH2 and HIF-1? were measured by western blot. GAPDH was used as a loading control.

## Abbreviations

CRC: colorectal cancer
EZH2: enhancer of zeste 2
HIF-1: hypoxia-inducible factor 1
NaPi2b: type II Na/Pi co-transporter
NC: negative control
qPCR: quantitative real-time RT-PCR analysis
ROS: reactive oxygen species
WT: wild-type
