# Supplementary material for: The SLC34A2-ROS-HIF-1-induced up-regulation of EZH2 expression promotes proliferation and chemo-resistance to apoptosis in colorectal cancer
Source: Biosci Rep. 2019 May 21;39(5):BSR20180268. doi: 10.1042/BSR20180268 (PMC6527931; doi:10.1042/BSR20180268)
Supplement: Supplementary file 1 [file bsr20180268_Supp1.pdf]

Supplemental Information

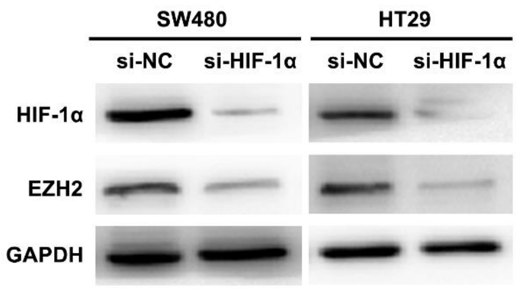

Figure S1. The expression of EZH2 and HIF-1α were detected by western blot in SW480 and HT29 cells infected with si-*HIF-1α* or si-NC. GAPDH was used as a loading control.

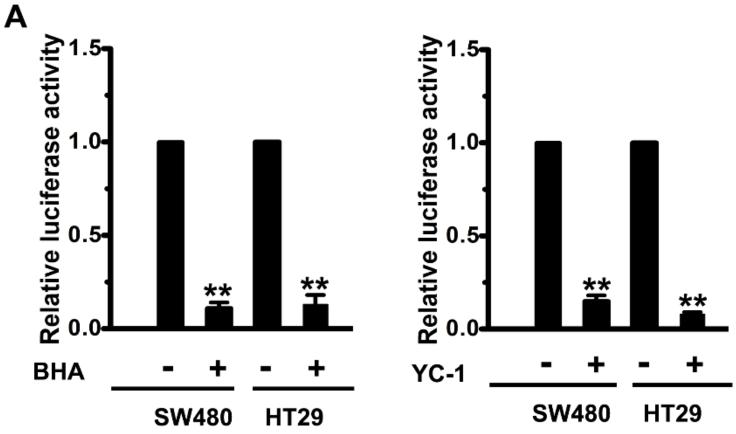

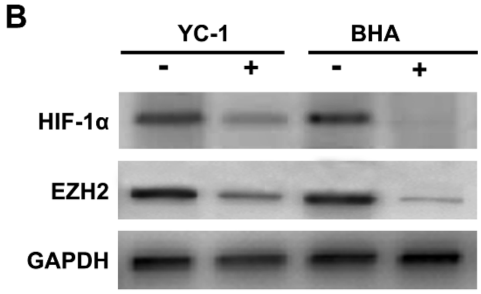

Figure S2. The effect of YC-1 and BHA were tested on SW480 and HT29 cells. (A) Luciferase activity was assayed in cells treated with or without YC-1 or BHA. Note: Columns, mean of three individual experiments; SD, **, P < 0.01. (B) SW480 cells were treated with or without YC-1 or BHA. The protein levels of EZH2 and HIF-1α were measured by western blot. GAPDH was used as a loading control.
